# Alkaline Stress Causes Changes in Polyamine Biosynthesis in *Thermus thermophilus*

**DOI:** 10.3390/ijms232113523

**Published:** 2022-11-04

**Authors:** Teruyuki Kobayashi, Akihiko Sakamoto, Keiko Kashiwagi, Kazuei Igarashi, Toshiyuki Moriya, Tairo Oshima, Yusuke Terui

**Affiliations:** 1 Faculty of Pharmacy, Chiba Institute of Science, Chiba 288-0025, Japan; 2 Amine Pharma Research Institute, Innovation Plaza at Chiba University, Chiba 260-0856, Japan; 3 Graduate School of Pharmaceutical Sciences, Chiba University, Chiba 260-8675, Japan; 4 Institute of Environmental Biology, Kyowa-Kako, Tokyo 194-0035, Japan

**Keywords:** polyamine, *Thermus thermophilus*, alkaline stress, cell growth

## Abstract

An extreme thermophile, *Thermus thermophilus*, produces 16 different polyamines including long-chain and branched-chain polyamines. The composition and content of polyamines in the thermophile cells change not only with growth temperature but also with pH changes. In particular, cell growth decreased greatly at alkaline medium together with significant changes in the composition and content of polyamines. The amounts of tetraamines (spermine and its homologs) markedly decreased at alkaline pH. Thus, we knocked out the *speE* gene, which is involved in the biosynthesis of tetraamines, and changes of composition of polyamines with pH changes in the mutant cells were studied. Cell growth in the Δ*speE* strain was decreased compared with that of the wild-type strain for all pHs, suggesting that tetraamines are important for cell proliferation. Interestingly, the amount of spermidine decreased and that of putrescine increased in wild-type cells at elevated pH, although *T. thermophilus* lacks a putrescine synthesizing pathway. In addition, polyamines possessing a diaminobutane moiety, such as spermine, decreased greatly at high pH. We assessed whether the *speB* gene encoding aminopropylagmatine ureohydrolase (TtSpeB) is directly involved in the synthesis of putrescine. The catalytic assay of the purified enzyme indicated that TtSpeB accepts agmatine as its substrate and produces putrescine due to the change in substrate specificity at high pH. These results suggest that pH stress was exacerbated upon intracellular depletion of polyamines possessing a diaminobutane moiety induced by unusual changes in polyamine biosynthesis under high pH conditions.

## 1. Introduction

Polyamines have been found in almost all organisms and are essential for normal cell growth and proliferation [1,2]. They are mainly bound to nucleic acids and modulate the functions of RNA and DNA [3,4,5]. Polyamines contribute to cell growth and viability of bacteria under various stresses, such as heat shock, oxidative and acid stresses [6,7,8]. Indeed, *Escherichia coli* and *Salmonella* spp. increase the pH of their surroundings due to the secretion of polyamines against acidic conditions. These mechanisms are known to trigger the expression of a specific battery of genes (*speF*, *cadA*, *cadB* and *paeA*, etc.), and specific polyamines synthesized are involved in, for example, the blockage of porins and a decrease in membrane permeability [9,10].

An extreme thermophile, *Thermus thermophilus* can grow at 47–85 °C, with optimal growth temperature at around 70 °C, and grows between pH 6.5 and 9.0 [11]. This thermophile produces 16 different polyamines, including the standard polyamines and long-chain and branched-chain polyamines (unusual polyamines, structures of polyamines are shown in Figure S1) [12]. Our previous studies demonstrated that unusual polyamines stabilize the conformation of nucleic acids, protect nucleic acids against depurination, and are essential for cell growth at high temperatures [13,14,15]. In extreme environments, the level of pH tolerance appears to be an important factor associated with the survival strategy of thermophiles. To adapt to environmental pH change, thermophiles possess mechanisms of protection for maintaining cellular pH homeostasis, such as very high intracellular cation concentrations, transport functions and membrane protein stability [16,17]. In particular, a high concentration of monovalent cations is necessary to maintain enzymatic activities and cell osmotic pressure. However, *T. thermophilus* contains low concentrations of intracellular monovalent cations compared to other thermophiles [18]. Therefore, unusual polyamines produced by *T. thermophilus* might be implicated in the regulation of intracellular cation concentration in the thermophile against environmental pH change. Polyamines are thought to play major roles in cation balance, but the exact involvement of specific polyamines still needs to be explored. In addition, few studies have tested the importance of polyamines when thermophiles are suffering pH stress.

In this study, the changes of composition and content of polyamines at various pH conditions were examined using *T. thermophilus* HB8. We found that the presence of these polyamines is effective for survival during pH stress, and the composition of polyamines in the thermophile cells changes not only with growth temperature but also with pH. In particular, polyamines possessing a diaminobutane moiety greatly decreased in alkaline conditions. A significant amount of putrescine was also detected at high pH, although the synthetic pathway of putrescine is not known in this strain. Therefore, we assessed whether the changes of polyamine biosynthesis during environmental pH change are involved in pH stress.

## 2. Results

### 2.1. Changes in Composition of Intracellular Polyamines, Cell Growth and Viability with pH Change

**Figure 1:**
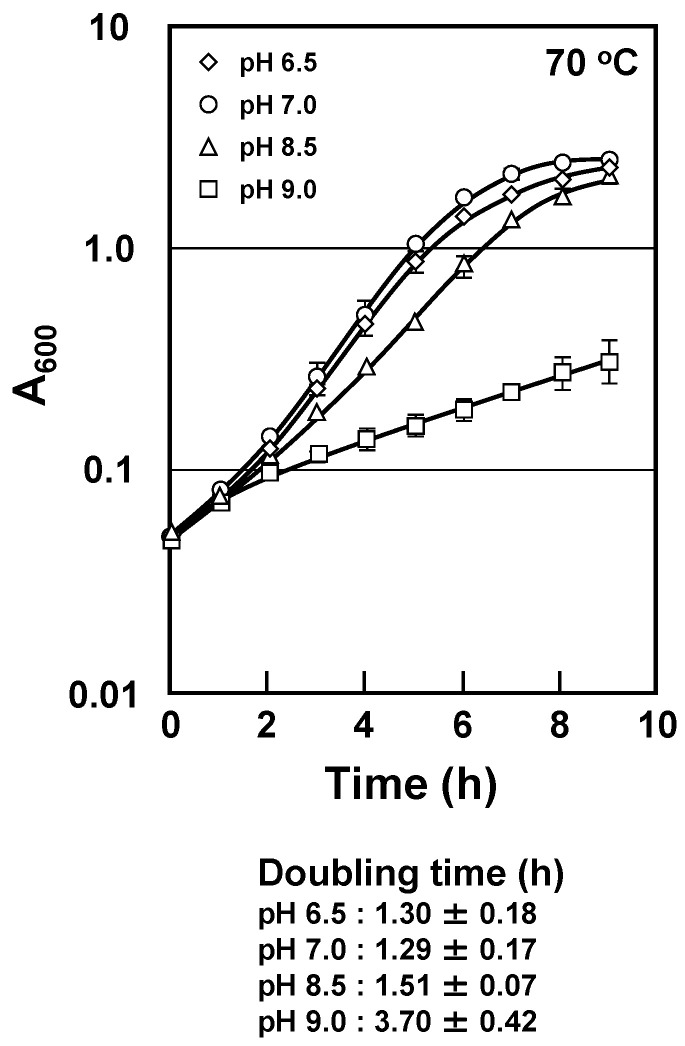
Cell growth of *T. thermophilus* HB8 grown at various pHs. Cell growth of *T. thermophilus* HB8 grown at pH 6.5 (◇), 7.0 (○), 8.5 (△) or 9.0 (□). The temperature was fixed at 70 °C. The results are shown as means ± standard errors, *n* = 3.

*T. thermophilus* can grow at pH 6.5–9.0 and the optimal growth temperature is around 70 °C. It is known that the optimum pH for growth of this strain was around 7.5 at room temperature; thus, it would be roughly pH 7 at 75 °C since the temperature dependent coefficient of the medium is −0.01 pH unit per degree [11]. Accordingly, we investigated cell proliferation of the wild-type strain in minimal medium at various pHs (pH = 6.5, 7.0, 8.5 and 9.0). As shown in Figure 1, cell growth was highest at pH 7.0, which is close to the optimal pH, followed by pH 6.5 and 8.5. However, the wild-type strain grew slowly at pH 9.0.

**Figure 2:**
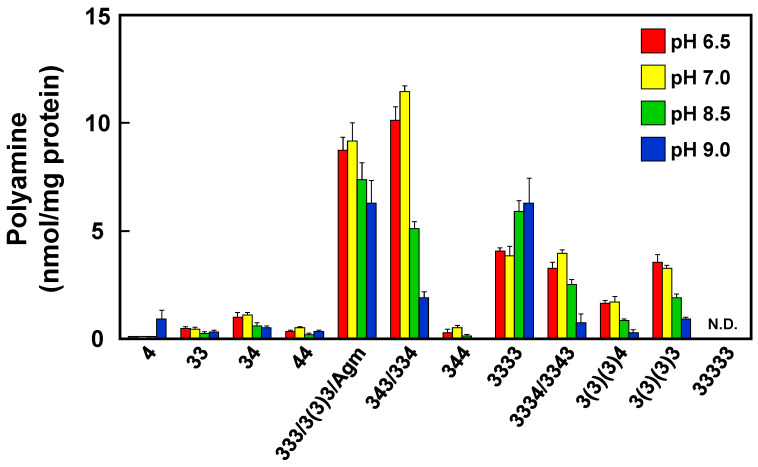
Polyamine content of *T. thermophilus* HB8 at various pHs. Polyamine content in cells harvested at an A_600_ of 0.5 was measured as described in Section 4. HPLC chromatograms are shown in Figure S2A. The contents are shown as means ± standard errors, *n* = 3. N.D., Not detected. Abbreviations are as follows: 4, putrescine; 33, norspermidine; 34, spermidine; 44, homospermidine; 333, thermine; 3(3)3, mitsubishine; Agm, agmatine; 343, spermine; 334, thermospermine; 344, homospermine; 3333, caldopentamine; 3334, homocaldopentamine; 3343, thermopentamine; 3(3)(3)4, *N*^4^-bis(aminopropyl)spermidine; 3(3)(3)3, tetrakis(3-aminopropyla)ammonium; 33333, caldohexamine.

Since polyamines are important for cell proliferation in many organisms, we measured the amount of intracellular polyamines at each pH (Figure 2). There were no significant changes in the total polyamine content and composition of the wild-type strain at pH 6.5 and 7.0. The total polyamine content and polyamine composition changed above pH 8.5. At pH 9.0, total polyamine content was decreased by about one third of that at pH 7.0. The amounts of tetraamines [spermine (343)/thermospermine (334)] and the derivatives such as homocaldopentamine (3334)/thermopentamine (3343) markedly decreased at elevated pH. At pH 9.0, the amounts of these polyamines possessing a diaminobutane moiety decreased to a tenth of that at pH 7.0. The other branched polyamines such as tris(3-aminopropyl)amine (3(3)3), tetrakis(3-aminopropyl)ammonium (3(3)(3)3) and *N*^4^-bis(aminopropyl)spermidine (3(3)(3)4) also decreased at elevated pH. Instead, an increased amount of putrescine was accumulated, whereas spermidine was decreased in the cells grown at pH 9.0. However, it is known that *T. thermophilus* lacks genes for known putrescine synthesizing enzymes, such as ornithine decarboxylase (*speC*) and agmatine iminohydrolase [19,20]. This result suggests that one or more other novel genes for putrescine biosynthesis could be responsible for the unusual synthesis of putrescine at high pH.

### 2.2. Changes in Polyamine Biosynthesis in T. thermophilus in Response to Environmental pH

**Figure 3:**
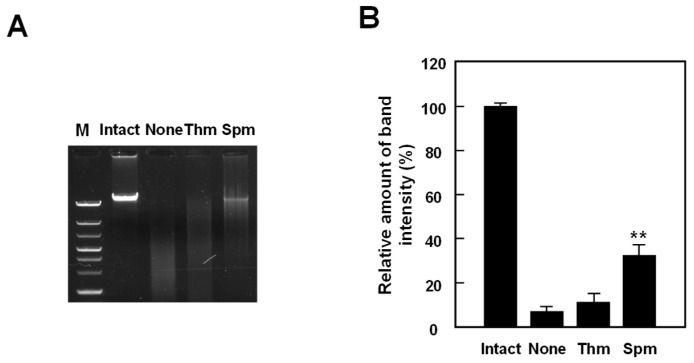
DSBs of *T. thermophilus* genome at high pH. (**A**,**B**) Degree of protection of wild-type genome against DSBs by thermine (Thm) and spermine (Spm). Experiments were conducted in the presence of 100 μM polyamines. The results are shown as mean ± standard errors of triplicate determinations. Student’s *t*-test was performed for the value of obtained in presence of polyamines versus its absence. **, *p* < 0.01.

At high pH, tetraamines such as spermine and its homologs which possess a diaminobutane moiety markedly decreased in the wild-type strain. We previously reported that polyamines protect nucleic acids against double-strand breaks (DSBs) and depurination [14,15]. To investigate the effect of these tetraamines on genome protection at high pH, we assessed the efficiency of polyamines on DSBs by electrophoresis of heat-treated samples on a neutral gel. As shown in Figure 3A,B, spermine (343), or its analog thermine (333), was mixed with the genome of the wild-type strain to study their protective effects against DSBs. Spermine protected DNA more effectively than thermine at pH 8.5. These results indicate that polyamines which have a diaminobutane moiety were necessary for protection of DNA at high pH.

**Figure 4:**
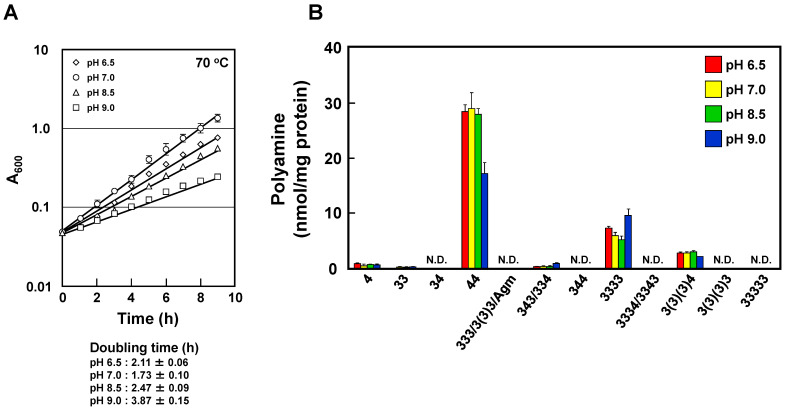
Cell growth and polyamine content of *T. thermophilus* HB8 Δ*speE* strain grown at various pHs. (**A**) Cell growth of *T. thermophilus* HB8 Δ*speE* strain grown at pH 6.5 (◇), 7.0 (○), 8.5 (△) or 9.0 (□). The temperature was fixed at 70 °C. The results are shown as means ± standard errors. (**B**) Polyamine content in cells harvested at an A_600_ of 0.5 was measured as described in Section 4. HPLC chromatograms are shown in Figure S2B. The contents are shown as means ± standard errors, *n* = 3. N.D., Not detected.

We have previously reported that polyamines possessing a diaminobutane moiety, such as spermidine and spermine, are synthesized by triamine/agmatine aminopropyltransferase (*speE*) [21]. To investigate the importance of the tetraamine biosynthetic pathway under pH stress, we disrupted the *speE* gene of *T. thermophilus*. As shown in Figure 4A, cell growth of the Δ*speE* strain significantly decreased at various pHs compared to that of the wild-type strain. Intracellular polyamine composition of the Δ*speE* strain grown in minimum medium at 70 °C was analyzed by HPLC (Figure 4B). Accumulation of agmatine seems to be small, whereas homospermidine significantly accumulated in the Δ*speE* strain. Tetraamines drastically diminished in the Δ*speE* strain. A small amount of putresine was also detected, but other polyamines, especially long and branched polyamines, were undetectable. Because the amount of homospermidine (44) increased greatly, there were no significant changes in the total polyamine content and polyamine composition of the Δ*speE* strain at various pHs compared to that of the wild-type strain. These results suggest that polyamine depletion brought about by a change to unusual polyamine biosynthesis led to pH sensitivity.

To more directly test the effects of polyamines, we measured intracellular and extracellular pH of the wild-type and the Δ*speE* strains cultured for 24 h at various pHs. The wild-type strain grown at pH 6.5 and 7.0, respectively, maintained neutral cytoplasmic pH when cultured for 24 h. However, the cytoplasm became more alkaline above pH 8.5 (Table S1).

### 2.3. Substrate-Specificity Change of T. thermophilus SpeB by External pH Change

At high pH, putrescine was detected in the wild-type strain and the Δ*speE* strain, although this bacterium lacks homologs of known putrescine synthesizing enzymes such as ornithine decarboxylase (*speC*) and agmatine iminohydrolase. In general, it is known that the alternative pathway of putrescine biosynthesis often seen in bacteria is hydrolysis of agmatine by agmatine ureohydrolase (*speB*). However, the enzyme encoded by *speB* in *T. thermophilus* does not accept agmatine as its substrate, but only accepts aminopropylagmatine at neutral pH [19]. However, the biosynthesis of putrescine at high pH could be catalyzed by aminopropylagmatine ureohydrolase (TtSpeB).

**Figure 5:**
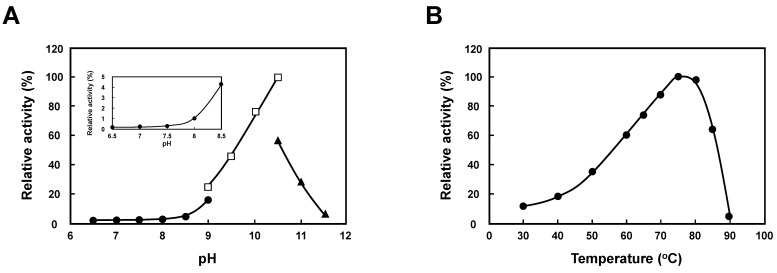
pH and temperature dependency of the enzymatic activity. The reactions were measured as described in Section 4. (**A**) pH dependency. Buffers used were Tris-HCl (pH 6.5–9), glycine-NaOH (pH 9–10.5), and CAPS-NaOH (pH 10.5–11.5). Reactions were performed for 1 h at 75 °C. (**B**) Temperature dependency. The reaction was performed in Tris-HCl (pH 8.5) for 1 h at 75 °C.

To confirm that TtSpeB uses agmatine as a substrate to produce putrescine at high pH, we purified TtSpeB to examine in vitro enzymatic activity. As shown in Figure 5A, the effect of pH on enzymatic activity was investigated over pH range of 6.5–11.5 at 75 °C, and the enzyme showed highest agmatine ureohydrolase activity at around pH 10.0. When the kinetic parameters were measured at different pHs, TtSpeB showed a Km for agmatine of 171 μM and *V*max of 1.22 mold/min/mg at pH 10.0. The apparent Km and *V*max values for agmatine of TtSpeB did not significantly change at pH 6.5–8.0. Relative activity at pH 8.5 was increased about 20-fold compared to that at pH 7.0. Thus, agmatine was accepted by TtSpeB at high pH, and putrescine was produced from agmatine. As shown in Figure 5B, the rate of the reaction was significantly increased by temperature elevation from 30 °C to 85 °C. The highest activity was detected at around 75 °C. These results indicate that TtSpeB accepts agmatine as its substrate and produces putrescine due to changes in substrate specificity at high pH. The substrate specificity of TtSpeB was significantly affected by pH. We have previously reported that spermidine is essential for the production of unusual polyamines [21]. These results indicate that the thermophile shows a significant defect in growth at high pH through the defect in the synthesis of long-chain and branched-chain polyamines which possess a diaminobutane moiety from putrescine.

## 3. Discussion

Polyamines are present in cells of many organisms and play important roles in cell proliferation and differentiation by interacting with nucleic acids. The standard polyamines in many organisms are spermine, spermidine and putrescine. However, thermophiles, including hyperthermophiles, produce unique polyamines, including long and/or branched-chain polyamines. Here, we showed that maintaining appropriate concentrations of intracellular polyamines enhances survival under pH stress. In particular, the composition, content of polyamines and cell growth markedly decreased in alkaline medium. These results indicate that polyamines are essential for cell proliferation of *T. thermophilus* against environment pH changes. When we look more closely at the changes in polyamine composition, spermine/thermospermine and homocaldopentamine/thermopentamine significantly decreased at high pH (Figure 2). Our previous studies reported that these polyamines decreased at high temperature. To investigate the effect of these polyamines on genome protection at high pH, we assessed the efficiency of polyamines on DSBs by electrophoresis of heat-treated samples on a neutral gel (Figure 3A,B). Spermine protected DNA more effectively than thermine at pH 8.5. These results indicate that polyamines which have a diaminobutane moiety were necessary for protection of DNA at high pH. The average distance between two adjacent phosphates of B-form DNA is 6.65 Å for the major groove. On the other hand, the N-N distances of diaminopropane and diaminobutane moieties of polyamines are 5.0 and 6.2 Å, respectively [22]. The finding is that the distance separating the charges, which is the number of methylene groups between amino groups, is important for the protection of DNA against DSBs at high pH. Triamine/agmatine aminopropyltransferase (TtSpeE), encoded by *speE*, is known to be a key enzyme for long-chain polyamine biosynthesis in *T. thermophilus*. In fact, cell growth and intracellular polyamines of the Δ*speE* strain markedly decreased (Figure 4A). Accumulation of agmatine seems to be small, and homospermidine significantly accumulated in the Δ*speE* strain (Figure 4B). It seems that homospermidine is produced from agmatine because it is present in the Δ*speE* strain. In other words, control of the concentrations of polyamines with aminobutyl groups is considered to be more important at high temperature and pH environments.

Many strains of the thermophilic bacterium *Thermus* are known to produce carotenoids [23]. Carotenoids are generated as scavengers of reactive oxygen species (ROS) in many bacteria for cellular protection and are also thought to be associated with membrane stabilization, which is considered essential for the growth of thermophilic bacteria at high temperatures [24]. When grown at pH 9.0, the strains produced less yellow pigment. The result suggests that the production of carotenoids decreases, and membranes become unstable, when polyamines are depleted. These findings suggest that polyamines may be involved in cell protection from hyperthermia and pH changes by regulating carotenoid biosynthesis and other processes.

Interestingly, the amount of spermidine decreased and that of putrescine increased in the cells at elevated pH, although *T. thermophilus* lacks a putrescine synthesizing pathway. It is known that putrescine significantly accumulates in many plants when potassium ions are deficient [25]. The small divalent cation, such as putrescine, may act as a surrogate for other monovalent or divalent cations in the cell to regulate cation balance, osmotic pressure, and pH. In general, it is known that the alternative pathway of putrescine biosynthesis that is often seen in bacteria is hydrolysis of agmatine by agmatine ureohydrolase (*speB*) [26]. However, the biosynthetic pathway from agmatine to putrescine is different from that in eukaryotes and other bacteria, putrescine is not synthesized from agmatine, because the enzyme encoded by *speB* in *T. thermophilus* does not accept agmatine as its substrate but only accepts aminopropylagmatine at neutral pH. It is known that the enzymatic activities, such as cellulase and D-amino acid oxidase in the alkaline range, vary depending on the size of substrate and product release involving a pH-dependent conformational change [27,28]. Therefore, we assessed whether the *speB* gene encoding aminopropylagmatine ureohydrolase is directly involved in the synthesis of putrescine at high pH. The catalytic assay of the purified enzyme indicated that TtSpeB accept agmatine as its substrate and produce putrescine due to changes in substrate specificity at high pH (Figure 5A,B). These results indicate that the thermophile is subject to a significant growth defect at high pH since long-chain polyamines which have a diaminobutane moiety are not well synthesized from putrescine.

In some mesophilic bacteria, putrescine and/or cadaverine, which are produced by triggering the expression of a specific battery of genes (*speF*, *cadA*, etc.) at low pH, neutralizes cytoplasmic pH [10,29]. The generation of these polyamines, followed by excretion via cotranscribed transporters, also neutralizes the acidic external environment. However, unusual polyamines such as putrescine and cadaverine were not detected in this thermophile at low pH. The results indicate that this bacterium may not encode a specific battery of genes involved in unusual polyamine biosynthesis in acid stress because the genome size of this thermophile is small compared to mesophilic bacteria. However, a minuscule amount of putrescine was produced by *T. thermophilus* when grown at neutral pH. The putrescine biosynthetic pathway in this bacterium has not yet been elucidated, and further research will be necessary.

## 4. Materials and Methods

### 4.1. Bacterial Strains and Culture Conditions

*T. thermophilus* HB8 (the wild-type strain) and the Δ*speE* strain were cultured overnight at 70 °C at 160 rpm in rich media of 0.8% tryptone, 0.4% yeast extract, 0.2% NaCl, 0.35 mM CaCl_2_ and 0.4 mM MgCl_2_, and were grown until A_600_ reached 1.0. Then, cell culture was started at an A_600_ = 0.05, and growth was monitored at 70 °C by measuring A_600_ in synthetic medium [30]. If necessary, the pH of the media was adjusted by the addition of 1N HCl or NaOH. The pH of media and cultures were measured at the corresponding growth temperature with a pH Meter S20 SevenEasy (Mettler-Toledo, Columbus, OH, USA). The pH value in the cells was determined as described previously [31]. Briefly, cultured cells were harvested by centrifugation and resuspended in ultrapure water at 5 times of the wet weight of the cells. The cells were then disrupted by sonication. After removing the cell debris by centrifugation, the supernatant was used for pH measurement.

### 4.2. Polyamine Analysis

The wild-type strain and/or Δ*speE* stain were cultivated in synthetic media at pH 6.5, 7.0, 8.5 or 9.0 until A_600_ = 0.5 and then harvested. Cells were disrupted in 15% trichloroacetic acid by sonication, for high performance liquid chromatography (HPLC) (HITACHI, Tokyo, Japan) analysis. The mixture was centrifuged, and the supernatant was used for HPLC analysis. HPLC analysis was carried out as described previously [15]. Protein content was determined using a Bradford Assay kit (Bio-Rad, Hercules, CA, USA).

### 4.3. Disruption of SpeE

Deletion of *speE* gene (TTHA0824) in *T. thermophilus* HB8 was performed as described previously [19,32]. For construction of the deletion plasmid, the gene encoding a kanamycin-resistant gene (*htk*) and both the upstream and downstream genomic regions flanking *speE* were amplified by polymerase chain reaction (PCR). Two pairs of primers: P1, 5′-GTCCTAGGTACCAAGGACCAGCTCAA-3′/P2, 5′-GTACATCCCGTAGTCCATATGT-CCTCC-3′, and P3, 5′-CTTACAAGCTGCAGCCTCTCCGGCAAG-3′/P4, 5′-GAAGAGGAGCGGCCGCTAATCGTAG-3′. These DNA fragments were assembled and inserted into KpnI-NdeI and PstI-NotI digested pBHTK plasmid [32]. The DNA mixture was used for the transformation of *E. coli* DH5α, and the plasmid was extracted from the kanamycin-resistant cells. The nucleotide sequence of the plasmids was confirmed with a 3130 Genetic Analyzer (Thermo Fisher Scientific, Waltham, MA, USA). To obtain a *speE* gene-disrupted strain of *T. thermophilus* HB8, the thermophile host was genetically transformed as described previously [19]. *Thermus* genomic DNA was extracted using the MonoFas^®^ gDNA Bacteria Extraction Kit VII (ANIMOS Inc., Saitama, Japan) according to the manufacturer’s instructions. All genetic recombinations were confirmed by PCR.

### 4.4. Assay of Double Strand Breaks

Double strand breaks analysis was carried out as described previously, with slight modifications [15]. Briefly, the reaction mixture contained 25 mM hydroxyethylpiperazine ethansulfonic acid (Hepes) buffer (pH 8.5) and 500 ng of *Thermus* genomic DNA was incubated at 95 °C for 50 min.

### 4.5. Expression and Purification of SpeB Protein

To make a *speB* (TTHA1129) expression plasmid (pET21speBHis), the gene was amplified by PCR using *T. thermophilus* HB8 genomic DNA as a template and two primers P5, 5′-AAGAATTCCATATGCGCCTCGTCTTCGGCGAAAAG-3′ and P6, 5′-AATTAAGCTTAATGTGGTCCACCTCCCGGGAAAG-3′. The DNA fragment was assembled and inserted into NdeI and HindIII digested pET21b plasmid (Merck, Germany). The nucleotide sequence of the plasmid was confirmed with a 3130 Genetic Analyzer (Thermo Fisher Scientific, Waltham, MA, USA). *E. coli* BL21(DE3) harboring pET21speBHis was grown in LB medium supplemented with 100 μg/mL ampicillin until A_600_ = 0.4 and then induced with 0.4 mM isopropyl β-D-1-thiogalactopyranoside (IPTG) at 20 °C. After 4 h of incubation, cells were harvested by centrifugation and resuspended in 20 mM Tris-HCl (pH 8.0), 20 mM MgCl_2_ and 10 mM MnCl_2_ at one-twentieth the volume of the culture medium. The cells were then disrupted by sonication. After removing the cell debris by centrifugation, the cell extract was heated at 70 °C for 20 min and the denatured protein was removed by centrifugation. The supernatant was diluted 4-fold with 20 mM Tris-HCl (pH 8.0) and applied to a Ni-NTA agarose column (QIAGEN, Venlo, The Netherlands), preequilibrated with the buffer A (50 mM Tris-HCl (pH 8.0), 0.3 M NaCl) containing 10 mM imidazole. The column was further washed with buffer A containing 30 mM imidazole. Elution was then performed using buffer A containing 300 mM imidazole. Purification efficiency was evaluated by sodium dodecyl sulfate-polyacrylamide gel electrophoresis. The purified His-tagged SpeB was then dialyzed against storage buffer (20 mM Tris-HCl (pH 8.0), 5 mM MgCl_2_, 1 mM DTT) and stored at 4 °C. Protein content was determined using a Bradford Assay kit (Bio-Rad, Hercules, CA, USA).

### 4.6. Enzymatic Reactions

The enzymatic reaction was performed as described previously [19]. To determine Km, *V*max and reaction products of SpeB, a reaction mixture consisting of 50 mM Tris-HCl (pH 10.0), 10 μM MnCl_2_, 0.005–1 mM agmatine and purified SpeB in a final volume of 100 μL was incubated for 1 h at 75 °C. The products of the enzymatic reaction were analyzed by HPLC as described in “Polyamine Analysis”.

## Data Availability

Not applicable.

## Supplementary Materials

The following supporting information can be downloaded at: https://www.mdpi.com/article/10.3390/ijms232113523/s1, Figure S1: Structures of polyamines in *T. thermophilus* HB8, Figure S2: HPLC chromatograms of polyamine analysis, Table S1: Extra- and intracellular pH of *T. thermophilus* HB8 and Δ*speE* strain grown at various pHs.
