# Supplementary material for: Alkaline Stress Causes Changes in Polyamine Biosynthesis in Thermus thermophilus
Source: Int J Mol Sci. 2022 Nov 4;23(21):13523. doi: 10.3390/ijms232113523 (PMC9654539; doi:10.3390/ijms232113523)
Supplement: Supplementary file 1 [file ijms-23-13523-s001.zip › Thermus pH Supplementary Materials_221102.pdf]

Figure S1

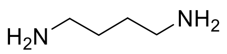

Putrescine (Put, 4)

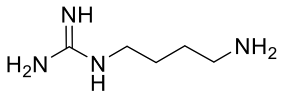

Agmatine (Agm)

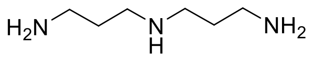

Norspermidine (Nspd, 33)

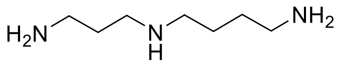

Spermidine (Spd, 34)

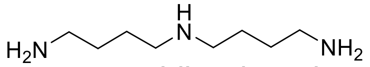

Homospermidine (Hspd, 44)

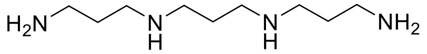

Thermine (Thm, 333)

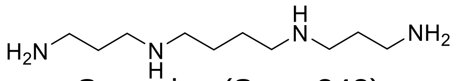

Spermine (Spm, 343)

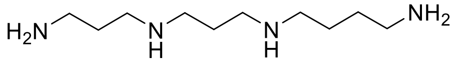

Thermospermine (Tspm, 334)

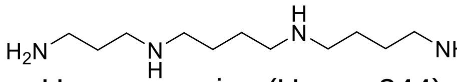

Homospermine (Hspm, 344)

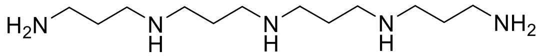

Caldopentarmine (Cdp, 3333)

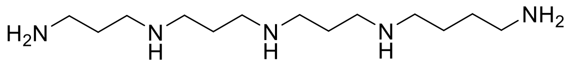

Homocaldopentarmine (Hcdp, 3334)

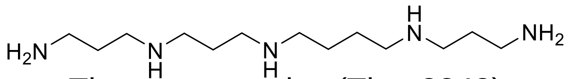

Thermopentamine (Thp, 3343)

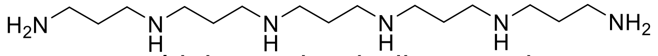

Caldohexamine (Cdh, 33333)

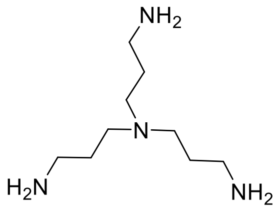

Tris(3-aminopropyl)amine
(Mitsubishine)
(Mb, 3(3)3)

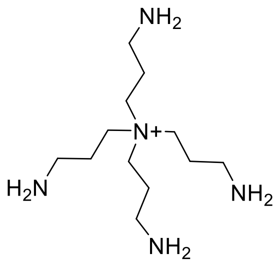

Tetrakis(3-aminopropyl)ammonium
(Taa, 3(3)(3)3)

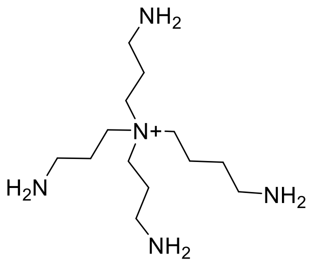

N[4]-bis(aminopropyl)spermidine
(3(3)(3)4)

Figure S2

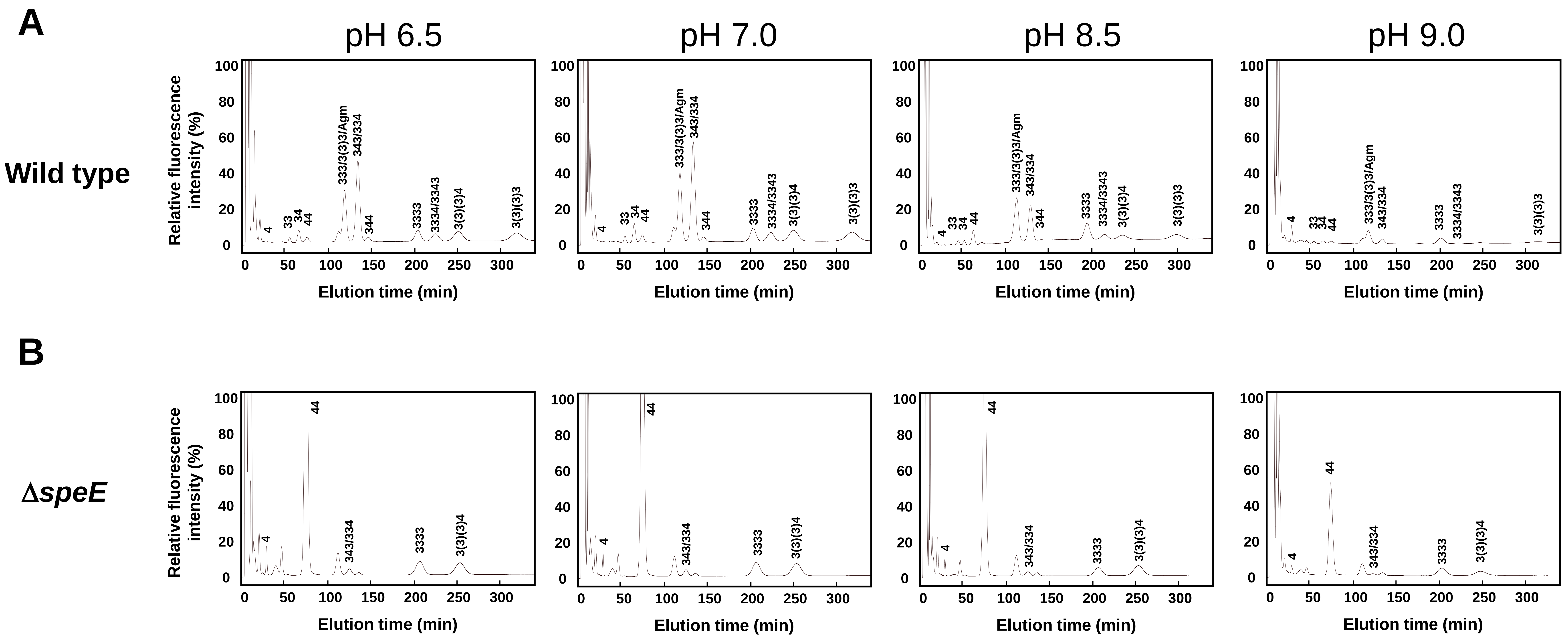
A
B
pH 6.5
pH 7.0
pH 8.5
pH 9.0
Wild type
ΔspeE
Relative fluorescence intensity (%)
Elution time (min)
4
33
34
44
333/3(3)3/Agm
343/334
344
3333
3334/3343
3(3)(3)4
3(3)(3)3

Table S1

Start pH	Wild type (A_{600} = 0.5) Extracellular pH	Wild type (A_{600} = 0.5) Intracellular pH	Δ*speE* (A_{600} = 0.5) Extracellular pH	Δ*speE* (A_{600} = 0.5) Intracellular pH
6.5	6.65±0.05	6.60±0.01	6.83±0.03	6.67±0.03
7.0	6.95±0.05	6.65±0.05	7.10±0.10	6.75±0.05
8.5	8.20±0.10	7.40±0.01	8.30±0.01	7.40±0.01
9.0	8.63±0.09	8.40±0.01	8.67±0.09	8.40±0.01
